# Knowledge, attitude, and practice of antenatal care providers about oral health care for pregnant women: a cross-sectional survey study in Shanghai

**DOI:** 10.3389/fpubh.2026.1807833

**Published:** 2026-06-22

**Authors:** Tingting Pan, Wenqi Hu

**Affiliations:** Dental Disease Prevention and Treatment Institute of Huangpu District, Shanghai, China

**Keywords:** antenatal care providers, cross-sectional study, health knowledge, attitude, practice, oral healthcare, pregnant women

## Abstract

**Introduction:**

This study investigated the knowledge, attitude, and practice (KAP) of obstetrician-gynecologists (OB-GYNs), nurses, and family physicians regarding oral healthcare for pregnant women.

**Methods:**

A cross-sectional study was conducted in Shanghai, China, using an electronic questionnaire distributed to 770 OB-GYNs, obstetrics/gynecology nurses, and family physicians in Huangpu District.

**Results:**

The 459 respondents (59.6% response rate) showed suboptimal knowledge, especially about medication and fluoride safety during pregnancy, and inconsistent oral health practices such as infrequent fluoride toothpaste recommendations and limited comprehensive counseling, despite generally positive attitudes toward integrating oral care into routine antenatal services. Over 80% of OB-GYNs and family physicians reported routinely giving oral health advice, and over 82% of nurses and family physicians advised on specific hygiene practices, such as brushing and rinsing. A key finding was that a minority of participants across all professions (19.2–34.5%) considered oral care solely the duty of dentists.

**Conclusion:**

This study identified knowledge gaps and limitations in specific oral health practices among antenatal care providers in Shanghai, especially among non-OB-GYN providers, despite generally positive attitudes. These findings indicate that strengthening providers’ knowledge and selected practice behaviors may be necessary to better support perinatal oral health services.

## Introduction

Changes in metabolism and lifestyle habits during pregnancy increase pregnant women’s susceptibility to oral diseases (1). Periodontitis in pregnancy is a preventable and treatable risk factor for adverse pregnancy outcomes such as preeclampsia, preterm labor, and low birth weight babies (2–4). Non-surgical periodontal treatments during pregnancy have been associated with an increased rate of preterm births (5). In addition, maternal oral health is associated with the onset of early childhood caries (ECCs), and receiving oral health care during pregnancy is a protective factor against ECCs (6–8). Therefore, oral health care during pregnancy is critical for maternal oral health, preventing adverse pregnancy outcomes and reducing the incidence of ECCs in infants and children (9).

Nevertheless, a review of worldwide survey reports suggested the underutilization of oral health care services during pregnancy among pregnant women (10). A study reported low consultation rates for oral diseases and even lower rates of women actively seeking preventive oral health care (11). A cohort study of 7,014 pregnant women in China showed that 35% of women in early pregnancy had never had an oral examination (12). Another survey in China showed that 41.86 and 36.34% of the women had received oral health care guidance from a dentist or obstetrician, respectively (13). A 2020 survey of pregnant women at an obstetrics and gynecology hospital in Shanghai found that 20.1% underwent pre-pregnancy oral examination, and the rate of correct tooth brushing was 49.6% (14).

Knowledge, attitude, and practice (KAP) studies are useful tools for investigating knowledge gaps, misconceptions, and misunderstandings that can hamper the correct performance of a specific topic within a specific population (15, 16). KAP studies are particularly useful for identifying barriers to optimal practice in a specific health-related topic, thereby informing the design of education interventions to improve practice. Studies have shown that mothers and children who receive oral health care services from a multidisciplinary team during pregnancy have better oral health status and higher scores for oral health care knowledge and behaviors (13, 17). Prenatal care providers, including obstetricians, gynecologists, nurses, and family physicians, have more frequent contact with pregnant women than dentists and represent a valuable resource for providing prenatal oral health knowledge and services (18). Prenatal care providers could promote maternal oral health (19) and increase the frequency of oral disease visits by providing oral health education, risk assessment, and referrals (20). The US National Maternal and Child Oral Health Resource Center published a resource guide for oral health care during pregnancy, suggesting that it is essential for prenatal care health professionals to provide pregnant women with appropriate and timely oral health care, including oral health education (21). The National Health Commission of the People’s Republic of China issued the Oral Health Action Program (2019–2025) in 2019 and emphasized that oral health knowledge should be a priority in premarital medical check-ups, maternal health management, and maternity school curricula, and that the healthcare providers should give full play to the synergistic role between maternal and child health care and dental institutions.

Although previous studies on women’s (22–27) and healthcare providers’ (28–30) oral health KAP have been conducted worldwide, there are no systematic surveys on the KAP of antenatal care providers regarding oral health care for pregnant women in Shanghai (China). One study examined dentists’ KAP regarding pregnant women’s oral health (29). Therefore, this survey aimed to examine the KAP of antenatal care providers in Huangpu District (Shanghai, China) toward oral health care during pregnancy and analyze the factors influencing KAP.

## Materials and methods

### Study design and participants

This cross-sectional survey enrolled 770 antenatal care providers, including family physicians in community hospitals, obstetricians and gynecologists (OB-GYN), and nurses in obstetrics and gynecology hospitals and in a maternal and child health institute in Huangpu District, Shanghai, from December 2020 to August 2021. This district-level setting reflects a typical urban Chinese maternal health service structure, where most pregnant women receive antenatal care through a combination of community-based family physicians and hospital-based obstetric services. The study was approved by the ethics board of Shanghai Huangpu District Dental Disease Prevention and Treatment Institute (approval #20201208–01). Informed consent was obtained from all participants before they completed the survey.

The inclusion criteria were 1) a family physician in community hospitals, OB-GYN, or a nurse in an obstetrics and gynecology hospital or maternal and child health institute, 2) with professional certification, and 3) working for at least 1 year in Huangpu District, Shanghai. Healthcare providers on leave, irrespective of the reason, were excluded.

### Study setting

Shanghai is a large metropolitan municipality in eastern China with over 24 million residents and a predominantly urban population, supported by a tiered healthcare system that includes community health centers and tertiary hospitals. Huangpu District, located in central Shanghai, is a densely populated urban district with an established network of community hospitals and tertiary obstetrics and gynecology centers that provide routine antenatal care. In this context, family physicians in community hospitals, obstetricians and gynecologists, and obstetric nurses are the main providers of prenatal care and represent the key professional groups responsible for delivering oral health information and referrals to pregnant women.

### Questionnaire

The questionnaire was originally developed by the research team using commonly applied KAP frameworks and previously published instruments on oral health in pregnancy by George et al. (31) and Da Costa et al. (32), and items were selected and tailored to routine practice and organizational characteristics of medical institutions in Shanghai. Because the instrument was designed specifically for this local context and not adapted from a foreign-language tool, formal cross-cultural translation and back-translation were not undertaken. The content validity and contextual appropriateness of the instrument were evaluated by three experts in dental public health, who critically reviewed the clarity of expression, conceptual relevance, and overall comprehensiveness of all items prior to its implementation.

The questionnaire covered four domains: (1) knowledge about perinatal oral health, (2–3) attitudes and practices related to oral health care for pregnant women, and (4) barriers to practice. Demographic data, including personal and training information, were also collected from the participants.

The survey design included multiple-choice questions (true/false/not sure) in the knowledge domain. Each correct answer received 1 point and each incorrect answer 0, yielding a maximum score of 12. A five-point Likert scale was adopted in the attitude domain, with the five items scored from 1 to 5, yielding a total score of 5–25. Questions regarding practice used a scale of always, sometimes, and never, scoring 3, 2, and 1, respectively, for a total maximum score of 27.

### Data collection and quality control

The medical departments of each participating hospital disseminated the anonymous electronic questionnaire (Appendix. Questionnaire) to eligible antenatal care providers (introduced earlier via on-site meetings and WeChat presentations), and responses were collected without identifying information. During the introduction sessions, participants were informed about the purpose, content, and voluntary nature of the study, and were allowed to ask questions and seek clarification. For the electronic questionnaire, an introductory page was provided at the beginning, outlining the study information and consent statement. Participants could proceed to complete the questionnaire after indicating their agreement to participate. Participation was entirely voluntary, and respondents could withdraw at any time during the process. The online questionnaire was delivered to respondents via the medical departments of each hospital. The questionnaire was completed anonymously and voluntarily. The purpose of the survey was introduced at the beginning, and respondents could withdraw from it at any time. One questionnaire could be submitted per IP address. Questionnaires could be submitted only if all questions were answered. Questionnaires with an obvious filling pattern (e.g., all first choices) were considered invalid.

### Sample size

The calculation of the sample size was based on the following formula employed for cross-sectional studies (33):


$$n={\left(\frac{Z_{1-\frac{\alpha }{2}}}{\delta }\right)}^{2}\times p\times (1-p)$$

where n denotes the sample size. p was set to 0.5 to achieve the maximum sample size. a refers to the type I error rate, which was set to 0.05. Subsequently, $Z_{1-\frac{\alpha }{2}}$ was 1.96. *δ* represents the effect sizes between groups, which was determined as 0.06, and at least 267 participants were required. Considering a 15% non-response rate, 315 participants were required.

### Statistical analysis

The data were analyzed using SPSS 25.0 (IBM, Armonk, NY, United States). Categorical data were presented as n (%) and analyzed using the chi-squared test or Fisher’s exact test. The continuous data were tested for normal distribution using the Kolmogorov–Smirnov test. The non-normally distributed continuous data were summarized as medians and interquartile ranges (IQRs) and analyzed using the Mann–Whitney U test. Variables with *p* < 0.05 in univariable analyses were included in multivariable logistic regression models to identify factors independently associated with practice scores. Two-sided *p*-values <0.05 were considered statistically significant.

## Results

### Demographics

A total of 770 eligible individuals were invited to participate, including 158 obstetrician-gynecologists (OB-GYNs; 27 from secondary hospitals and 131 from tertiary hospitals), 190 family physicians (all from primary hospitals), and 422 nurses (38 from secondary hospitals and 384 from tertiary hospitals). Of these, 459 completed questionnaires were returned, yielding a response rate of 59.6%. None were excluded because they had an obvious filling pattern. The sample included all three main provider groups involved in antenatal care in Huangpu District, OB-GYNs and obstetrics/gynecology nurses from the participating hospitals, and family physicians from community health centers, and their distribution by gender, age, education, and professional title reflected the predominantly female, mid-career workforce in these settings (Table 1). Nurses, OB-GYNs, and family physicians accounted for 51.0, 25.1, and 24.0% of the participants, respectively. The majority of the participants were female, aged 31–40 years, with a bachelor’s degree, residents, and had no training in oral care (Table 1).

**Table 1:** Demographic characteristics and KAP scores of the participants (*N* = 459).

Index	OB-GYN (*n* = 115) n (%)	Family physicians (*n* = 110) n (%)	Nurses (*n* = 234) n (%)
Gender			
Male	12 (10.4)	31 (28.2)	15 (6.4)
Female	103 (89.6)	79 (71.8)	219 (93.6)
Age (years) at questionnaire completion			
≤30	41 (35.7)	6 (5.5)	67 (28.6)
31–40	51 (44.3)	38 (34.5)	109 (46.6)
41–50	19 (16.5)	54 (49.1)	38 (16.2)
>50	4 (3.5)	12 (10.9)	20 (8.5)
Education			
Associate	2 (1.7)	3 (2.7)	77 (32.9)
Bachelor	48 (41.7)	86 (78.2)	152 (65)
Master	34 (29.6)	21 (19.1)	5 (2.1)
PhD	31 (27.0)	0 (0)	0 (0)
Title			
Resident	49 (42.6)	5 (4.5)	121 (51.7)
Attending physician	32 (27.8)	87 (79.1)	89 (38)
Professor	34 (29.6)	18 (16.4)	24 (10.3)
Hospital level			
Primary	0 (0)	110 (100)	0 (0)
Secondary	17 (14.8)	0 (0)	24 (10.3)
Tertiary	98 (85.2)	0 (0)	210 (89.7)
Received training experience in oral care			
Never	62 (53.9)	62 (56.4)	115 (49.1)
Sometimes	38 (33)	37 (33.6)	100 (42.7)
Always	15 (13)	11 (10)	19 (8.1)
Knowledge score, M (IQR)	9 (3)	5 (4)	6 (4)
Attitude score, M (IQR)	21 (3)	21 (4)	21 (4)
Practice score, M (IQR)	24 (6)	24 (5)	23 (6)

Data are presented as n (%) or median (interquartile range, IQR), as appropriate. KAP, knowledge, attitude, and practice; OB-GYN, obstetrician-gynecologist; IQR, interquartile range. Training experience was based on self-reported previous professional training related to oral health care for pregnant women and was categorized as never, sometimes, or always. Knowledge score was calculated from 12 items, with 1 point assigned for each correct answer and 0 points assigned for an incorrect or “not sure” response; total score range, 0–12. Attitude score was derived from five items assessing attitudes toward oral health care during pregnancy, each scored on a five-point Likert scale from 1 to 5. The total score ranges 5–25, and higher total scores indicate more positive attitudes. Practice score was derived from 9 items assessing self-reported oral health care practices for pregnant women. The total score ranges 9–27, and higher scores indicate better practice.

### Knowledge

OB-GYNs demonstrated significantly higher overall knowledge scores compared with family physicians and nurses (median [IQR]: 9 [3] vs. 5 [4] and 6 [4], respectively; *p* < 0.001; Table 1). Across all provider categories, OB-GYNs achieved the highest median knowledge scores, followed by nurses and family physicians (Table 1). Among OB-GYNs, higher educational attainment was significantly associated with greater knowledge scores (*p* < 0.001; Supplementary Table S1). Among family physicians, both higher education and prior training experience were associated with higher knowledge scores (*p* = 0.031 and *p* = 0.003, respectively; Supplementary Table S2). Among nurses, younger age, working in higher-level hospitals, and training experience were associated with higher knowledge scores (*p* < 0.001, *p* = 0.020, and *p* = 0.037, respectively; Supplementary Table S3). Fewer than 60% of OB-GYNs correctly answered items on the safety of fluoride toothpaste use during pregnancy and the safety of ibuprofen in late pregnancy. Fewer than 60% of nurses responded correctly to several key items, including the association between periodontal disease and adverse pregnancy outcomes, the link between high maternal caries prevalence and child caries risk, the safe treatment period for oral disease in pregnancy, dental X-rays, fluoride toothpaste, acetaminophen and metronidazole use, and ibuprofen safety in late pregnancy. Fewer than 60% of family physicians answered correctly regarding dental X-rays, fluoride toothpaste, acetaminophen, cephalosporins, metronidazole, and ibuprofen safety in late pregnancy (Supplementary Table S4). Significant between-group differences were observed for all knowledge items except two: “Pregnant women with poor oral hygiene are more likely to develop gingivitis during pregnancy (YES)” and “Maternal caries may promote caries in the child (YES)” (Supplementary Table S4). Overall, these findings indicate that antenatal care providers’ knowledge of oral health during pregnancy is suboptimal, with marked gaps in key clinical topics (e.g., medication and fluoride safety) despite OB-GYNs scoring higher than nurses and family physicians.

### Attitudes

The attitude scores could vary between 5 (all items with a poor attitude) and 25 (all items with a positive attitude). Overall, the three provider groups had comparable attitude scores (21 (3), 21 (4), and 21 (4); *p* > 0.05; Table 1). Among OB-GYNs, male gender and older age were associated with higher attitude scores (*p* = 0.011 and *p* = 0.040, respectively; Supplementary Table S1), whereas among family physicians, older age was associated with higher attitude scores (*p* = 0.046; Supplementary Table S2). Among nurses, being 41–50 years old was associated with higher attitude scores (*p* = 0.008; Supplementary Table S3). At the item level, more than 80% of OB-GYNs, nurses, and family physicians agreed that oral health care should be part of routine prenatal care and that antenatal providers should counsel pregnant women on maternal and infant oral health. Significant between-group differences were observed for the item on the importance of maternal oral health for the infant’s oral health (*p* = 0.040; Supplementary Table S5). Taken together, the results suggest that antenatal care providers generally hold positive attitudes toward integrating oral health into prenatal care, although the perceived importance of maternal oral health for infant outcomes remains variable across professional groups.

### Practice

The practice scores could vary between 9 (all items never practiced) and 27 (all items always practiced). Overall, the three provider groups showed similar practice scores (24 (6), 24 (5), and 23 (6); *p* > 0.05; Table 1). Among OB-GYNs, older age and prior training experience were associated with higher practice scores (Mann–Whitney U test; *p* = 0.021 and *p* < 0.001, respectively; Supplementary Table S1), while among family physicians, training experience alone was associated with higher practice scores (Mann–Whitney U test; *p* < 0.001; Supplementary Table S2). Among nurses, working in secondary hospitals and having training experience were associated with higher practice scores (Mann–Whitney U test; *p* = 0.003 and *p* < 0.001, respectively; Supplementary Table S3). At the item level, more than 80% of OB-GYNs and family physicians reported that they always provided oral health advice when pregnant women encountered oral problems and advised them to brush their teeth in the morning and evening, and 84.2% of nurses always advised twice-daily toothbrushing, whereas 82.7% of family physicians always recommended rinsing after meals. Other oral health service behaviors, such as recommending fluoride toothpaste, were reported less frequently, with fluoride toothpaste recommendations being the least common practice across all three provider categories (Supplementary Table S6). In summary, while core counseling behaviors such as advising on toothbrushing and addressing oral problems are commonly reported, more comprehensive oral health practices (e.g., regular dental referrals and fluoride toothpaste recommendations) remain insufficient and appear strongly influenced by prior training and work context.

### Barriers

A lack of knowledge about oral health care for pregnant women was the most frequently cited barrier among OB-GYNs, nurses, and family physicians, affecting their oral health care practice. Among the participants, 24.3, 34.5, and 19.2% of OB-GYNs, family physicians, and nurses, respectively, chose the barrier “It’s the duty of dentists”. There were statistically significant differences in practice scores across the three participant categories for all barriers (chi-squared test; *p* < 0.05), except for “Legal risks associated with negative birth outcomes” (Table 2).

**Table 2:** Barriers affecting antenatal care providers in providing oral health advice to pregnant women.

Barriers	OB-GYN (*n* = 115)	Family physicians (*n* = 110)	Nurses (*n* = 234)	Chi-squared	*p*
Lack of time in providing advice about oral health care to pregnant women.	42 (36.5)	23 (20.9)	58 (24.8)	7.970	0.019
It’s the duty of dentists.	28 (24.3)	38 (34.5)	45 (19.2)	9.574	0.008
Lack of knowledge about oral health care for pregnant women.	91 (79.1)	77 (70.0)	192 (82.1)	6.468	0.039
Legal risks associated with negative birth outcomes	16 (13.9)	22 (20.0)	44 (18.8)	1.706	0.426
Lack of consensus among dentists on oral health care and treatment of pregnant women	67 (58.3)	49 (44.5)	164 (70.1)	21.001	<0.001
Pregnant women are not interested or have no need	30 (26.1)	38 (34.5)	114 (48.7)	18.079	<0.001

Data are presented as n (%). OB-GYN, obstetrician-gynecologist. The listed barriers refer to self-reported factors affecting the provision of oral health advice to pregnant women. *p* values were calculated for comparisons among the three professional groups.

### Factors influencing antenatal care providers’ oral health care practices for pregnant women

In the multivariable logistic regression models, among OB-GYNs, age 31–40 (vs. ≤30; OR = 3.4, 95% CI: 1.2–9.7, *p* = 0.026), master’s degree (vs. PhD; OR = 4.3, 95% CI: 1.1–16.9, *p* = 0.038), with training experience (OR = 5.3, 95% CI: 1.8–16.0, *p* = 0.003), and “it’s dentists’ duty” (OR = 0.1, 95% CI: 0.04–0.4, *p* < 0.001) were independently associated with the practice scores (Table 3). Among family physicians, age ≤30 (vs. 40–50; OR = 0.1, 95% CI: 0.02–0.9, *p* = 0.038), age 31–40 (vs. 40–50; OR = 0.3, 95% CI: 0.1–0.9, *p* = 0.029), and “it’s dentists’ duty” (OR = 0.2, 95% CI: 0.1–0.7, *p* = 0.007) were independently associated with the practice scores (Table 4). Among nurses, working in tertiary hospitals (vs. secondary; OR = 0.2, 95% CI: 0.04–0.7, *p* = 0.017), the knowledge scores (OR = 1.2, 95% CI: 1.1–1.4, *p* = 0.002), with training in oral care (OR = 2.7, 95% CI: 1.4–5.0, *p* = 0.002), and “it’s dentists’ duty” (OR = 0.5, 95% CI: 0.2–1.0, *p* = 0.048) were independently associated with the practice scores (Table 5).

**Table 3:** Multivariable analysis of factors associated with practice level among OB-GYNs (*n* = 115).

Variables	Group	B	S.E	Wald χ^2^	*p*	OR	95% CI for OR
Age (years)	≤ 30*						
	31–40	1.2	0.5	5.0	0.026	3.4	1.2–9.7
	41–50	1.7	1.0	3.1	0.080	5.6	0.8–37.7
	> 50	0.1	1.6	0.0	0.957	1.1	0.04–27.7
Education	PhD*						
	Master	1.5	0.7	4.3	0.038	4.3	1.08–16.9
	Associate & bachelor	0.5	0.6	0.6	0.428	1.6	0.5–5.0
Hospital level	Secondary*						
	Tertiary	−1.6	1.4	1.2	0.268	0.2	0.01–3.4
Training experience	No*						
	Yes	1.7	0.6	8.9	0.003	5.3	1.8–16.0
“It’s dentists’ duty”	No*						
	Yes	−2.2	0.6	12.3	<0.001	0.1	0.04–0.4

OB-GYN, obstetrician-gynecologist; OR, odds ratio; CI, confidence interval. The dependent variable was the practice score. Reference categories are shown in parentheses. ORs>1 indicate factors associated with better practice scores. ORs<1 indicate factors associated with poorer practice scores. *reference category; n indicates the number of participants included in the corresponding analysis.

**Table 4:** Multivariable analysis of factors associated with practice level among family physicians (*n* = 110).

Variables	Group	B	S.E	Wald χ^2^	*p*	OR	95% CI of OR
Age (years)	≤ 30	−2.1	1.0	4.3	0.038	0.1	0.02–0.9
	31–40	−1.3	0.6	4.8	0.029	0.3	0.09–0.9
	40–50*						
	> 50	−0.2	0.9	0.1	0.794	0.8	0.1–4.8
Training experience	No*						
	Yes	0.8	0.6	2.3	0.133	2.3	0.8–6.8
“It’s dentists’ duty”	No*						
	Yes	−1.4	0.5	7.2	0.007	0.2	0.09–0.7

OR, odds ratio; CI, confidence interval. The dependent variable was the practice score. Reference categories are shown in parentheses. ORs>1 indicate factors associated with better practice scores. ORs<1 indicate factors associated with poorer practice scores. *reference category; n indicates the number of participants included in the corresponding analysis.

**Table 5:** Multivariable analysis of factors associated with practice level among nurses (*n* = 234).

Variables	Group	B	S.E	Wald χ^2^	*p*	OR	95% CI of OR
Education	Associate*						
	Bachelor	0.6	0.3	3.5	0.060	1.9	1.0–3.5
	Master	1.1	1.2	0.9	0.335	3.1	0.3–30.6
Hospital level	Secondary*						
	Tertiary	−1.7	0.7	5.7	0.017	0.2	0.04–0.7
Attitude score		0.1	0.1	3.2	0.073	1.1	1.0–1.2
Knowledge score		0.2	0.1	9.1	0.002	1.2	1.1–1.4
Training experience	No*						
	Yes	1.0	0.3	9.4	0.002	2.7	1.4–5.0
“It’s dentists’ duty”	No*						
	Yes	−0.8	0.4	3.9	0.048	0.5	0.2–1.0

OR, odds ratio; CI, confidence interval. The dependent variable was the practice score. Reference categories are shown in parentheses. ORs>1 indicate factors associated with better practice scores. ORs<1 indicate factors associated with poorer practice scores. *reference category; n indicates the number of participants included in the corresponding analysis.

## Discussion

This cross-sectional study investigated the KAP of OB-GYNs, nurses, and family physicians in Huangpu District (Shanghai, China) toward oral healthcare for pregnant women. The results suggest a need to improve the KAP of oral health during pregnancy among OB-GYNs, family physicians, and nurses in Shanghai (China).

The median knowledge scores of OB-GYNs in this survey were significantly higher than those of family physicians and obstetrical/gynecological nurses (9 vs. 5 vs. 6), reflecting the lack of knowledge of family physicians and obstetrical/gynecological nurses about the oral health of pregnant women, similar to Boutigny et al. (34). Although OB-GYNs had higher knowledge scores in this survey, they also answered fewer specific questions correctly, such as the safety of fluoride toothpaste use in pregnant women (45.2%), the safety of dental X-rays during pregnancy (60.9%), and the fact that caries in mothers can increase the risk of caries in their children (67.0%). In a survey by Kobylinska et al. (35), OB-GYNs had correct perceptions of the safety of fluoride toothpaste and dental X-rays, and that maternal caries increased the risk of caries in the child (74.6, 62.2, and 49.2%). SteelFisher et al. (36) showed that 84% of OB-GYNs inquired about pregnancy-related medication safety information during pregnant women’s visits rather than before or after the visit, which may be one of the reasons for the low rate of correct responses by the majority of the respondents in this survey about the safety of commonly used medications in dentistry during pregnancy. In addition, the correct response rates of OB-GYNs and nurses regarding the safety of ibuprofen use in the third trimester were 14.8 and 19.7%, respectively, which were significantly lower than that of family physicians (30.9%) and may also be related to the relatively low use of ibuprofen in obstetrics and gynecology. On the other hand, compared with the low percentage of correct responses from family physicians (14.5%) regarding the safety of metronidazole use during pregnancy, the percentage of correct responses from OB-GYNs (59.1%) and nurses (38.9%) was relatively high, which may also be because metronidazole is more commonly used in outpatient obstetrics and gynecology clinics.

The participants were generally positive about oral health care during pregnancy. However, 46.1, 28.2, and 31.6% of OB-GYNs, family physicians, and nurses, respectively, opposed postponing oral treatments after delivery, indicating a clear division. It may be due to concerns about the health of the pregnant woman and the fetus, as well as a lack of understanding of the dental treatments. Nevertheless, a survey of OB-GYNs in Beijing showed that 72.9% of the respondents believed oral treatments were safe for healthy pregnant women (37). In a cross-sectional survey by George et al. (31), 86.1% of antenatal care providers opposed the statement that “pregnant women should receive emergency dental care only”. In a study by Paneer et al. (38), 90% of respondents denied recommending that pregnant women delay seeing a dentist. The American Dental Association (ADA) Dental Guidelines for Pregnancy clearly state that preventive, diagnostic, and restorative dentistry is safe throughout pregnancy (39, 40).

In this survey, the oral health care advice given to pregnant women by OB-GYNs, nurses, and family physicians all focused on providing referral advice when necessary, brushing teeth in the morning and evening, and rinsing after eating, while advice on regular oral exams, dental flossing and fluoride toothpaste use, and educating pregnant women about the importance of maternal and newborn oral health care was provided relatively infrequently and was correlated with relevant training experiences, suggesting the importance of enhanced training. In the survey by Then et al. (41), 69.7% of the respondents advised their patients to visit a dentist, and 63.6% referred their patients to a dentist during pregnancy.

In a survey conducted in Beijing, OB-GYNs identified the main barriers to providing oral health care services to pregnant women as the “lack of knowledge” (79.2%), “perceived to be the job of the dentist” (75.5%), “too busy in the clinic to have time” (74.3%), and “lack of consensus” (65.3%) (42). In a cross-sectional survey of antenatal care providers conducted by George et al. (31), ‘lack of time’ (59%) and ‘lack of consensus’ (56.7%) were the main barriers to the practice of oral health care services. In the present study, “lack of knowledge” and ‘lack of consensus’ (58.3%) were the main barriers to practice identified by the respondents of this survey. Lulu et al. (43) showed that a good understanding between dentists and antenatal care providers and the availability of professional service guidelines help antenatal care providers provide oral health services to pregnant women.

The multivariable logistic regression analysis indicated that the position that “it is the dentists’ duty” was a deterrent to participants’ oral health care practices. In contrast, the experience of training related to oral health care for pregnant women was a facilitator. In addition, 24.3, 19.2, and 34.5% of OB-GYNs, nurses, and family physicians, respectively, perceived that it is the dentists’ duty to advise pregnant women on oral health care in terms of responsibility orientation, which is consistent with the high median practice scores of the respondents (24 vs. 23 vs. 24), reflecting the good responsibility orientation of the respondents and the good practice orientation of the respondents in the present survey. Younger age was also associated with less effective practice in OB-GYNs and family physicians, which could reflect work experience.

Nevertheless, in light of these findings, interventions should extend beyond individual training to address system-level determinants of antenatal oral health care. First, clinical guidelines or consensus statements on oral health during pregnancy, jointly developed by obstetrics/gynecology, family medicine, nursing, and dental professional bodies, are needed to standardize recommendations on examination timing, safe procedures, and medication use. Second, integrating oral health into routine antenatal workflows (for example, through brief screening questions, electronic medical record prompts, and embedded referral pathways to dental services) could help translate positive attitudes into consistent practice. Third, structured interprofessional collaboration mechanisms, such as shared care pathways, joint perinatal-dental clinics, or regular case-based meetings, may facilitate coordination between antenatal care providers and dentists, as highlighted by several guidelines from various countries (21, 44–46). Finally, policy-level measures, such as incorporating oral health indicators into maternal health programs and performance frameworks, could incentivize institutions to prioritize antenatal oral health services.

Future research should move beyond descriptive KAP assessment to develop and evaluate targeted interventions for antenatal care providers, including training programs and system-level strategies that address the specific gaps identified in this survey. Multicenter or regional studies are also needed to determine whether similar KAP patterns and barriers exist in other Chinese settings and health care systems. In addition, longitudinal and mixed-methods studies could explore how improvements in providers’ KAP influence pregnant women’s oral health behaviors, service use, and perinatal oral health outcomes.

Given that training experience was consistently associated with higher knowledge and practice scores in all three professional groups, a structured train-the-trainer model may be particularly appropriate in Huangpu District. In such a model, selected OB-GYNs, nurses, and family physicians would receive focused training in antenatal oral health from district-level dental public health experts and tertiary hospital dentists, and subsequently deliver brief, recurrent training and case-based support to colleagues within their own facilities.

This study had limitations. The study was performed in a single district in Shanghai, limiting generalizability to other regions. The study was cross-sectional, preventing the evaluation of causality or the temporal evolution of KAP. Nevertheless, the present study could serve as a historical baseline for future intervention studies. All KAP studies are at risk of social desirability bias, in which participants may be tempted to answer what they know they should think or do rather than what they actually think or do (47, 48). No pilot study of the questionnaire was performed before the formal study to determine the questionnaire’s validity metrics. Finally, the study was conducted in a single urban district and achieved a 59.6% response rate; although respondents were broadly similar to the underlying antenatal workforce in terms of key demographics, the findings may not fully represent all providers in Shanghai or other regions.

## Conclusion

This survey showed that, although antenatal care providers in Huangpu District generally held positive attitudes toward integrating oral health into prenatal care, OB-GYNs had higher knowledge scores than nurses and family physicians, and important gaps persisted in specific topics and practices, particularly among non-OB-GYN providers. Targeted efforts to improve providers’ knowledge and selected oral health practices, therefore, appear warranted to strengthen antenatal oral health care. Beyond strengthening training for antenatal care providers, future efforts should prioritize developing clear clinical guidance, workflow-integrated oral health processes, and interprofessional and policy frameworks that embed oral health within routine maternal care.

## Data Availability

The original contributions presented in the study are included in the article/Supplementary material, further inquiries can be directed to the corresponding author.
