# Supplementary material for: Knowledge, attitude, and practice of antenatal care providers about oral health care for pregnant women: a cross-sectional survey study in Shanghai
Source: Front Public Health. 2026 Jun 22;14:1807833. doi: 10.3389/fpubh.2026.1807833 (PMC13333608; doi:10.3389/fpubh.2026.1807833)
Supplement: Supplementary file 1 [file Data_Sheet_1.docx]

**Questionnaire for antenatal care providers on oral health care for pregnant women**

Dear Participant,

Hello. We are members of the research team and sincerely invite you to participate in this questionnaire survey. This study aims to investigate antenatal care providers’ knowledge, attitudes, and practices regarding oral health care for pregnant women, in order to provide evidence for developing scientific and feasible health education and service-improvement strategies. This study has been approved by the Ethics Review Committee, and your participation is entirely voluntary.

Please complete the questionnaire according to your actual situation and true opinions. If you have any questions during the process, you may consult the researchers at any time.

This is an anonymous questionnaire survey and will not cause any physical or psychological harm to you. The questionnaire may collect basic information such as your gender, age, and occupation. All information will be used only for scientific research and will be kept strictly confidential.

You have the right to obtain information related to this study at any time. You may also withdraw before submitting the questionnaire. If you choose to withdraw, your data will not be included in the analysis.

Completion and submission of the questionnaire indicate that you have read and understood the above information and agree to participate in this study.

Thank you sincerely for taking the time to support this scientific research.

□ I have read and understood the above information, agree to participate in this study, and consent to the use of the collected data for scientific research.

1. **Demographics**

1.1 Gender: 1.Male 2.Female

1.2 Age Range in Years: 1.≤30 2.31-40 3.41-50 4.>50

1.3 Highest Qualification: 1.Associate 2.Bachelor 3.Master 4.Phd

1.4 Job Title: 1.Residency 2.Attending 3.Professor

1.5 Working Hospital Level: 1.First Level 2.Secondary Level 3.Tertiary Level

1.6 Occupation: 1.Obstetrician & Gynecologist 2.Nurse 3.Family Physician

1.7 Have you received any professional training on oral health care for pregnant women?

1.Never 2.Sometimes 3.Always

1. **Attitude about oral health care for pregnant women**

| Oral health care should be a part of prenatal care. | Strongly Agree | Somewhat Agree | Neutral | Somewhat Disagree | Strongly  Disagree |
| --- | --- | --- | --- | --- | --- |
| Maternal oral health affects infant oral health. | Strongly Agree | Somewhat Agree | Neutral | Somewhat Disagree | Strongly  Disagree |
| Antenatal care providers should advise pregnant women on maternal and infant oral health care information about maternal and infant. | Strongly Agree | Somewhat Agree | Neutral | Somewhat Disagree | Strongly  Disagree |
| Antenatal care providers should provide oral health advice to pregnant women at clinic visits. | Strongly Agree | Somewhat Agree | Neutral | Somewhat Disagree | Strongly  Disagree |
| Oral problems during pregnancy should be postponed until after delivery. | Strongly Agree | Somewhat Agree | Neutral | Somewhat Disagree | Strongly  Disagree |

1. **Knowledge about oral health care during pregnancy**

| Periodontal disease in pregnant women may lead to adverse pregnancy outcomes. | True | False | Not Sure |
| --- | --- | --- | --- |
| Pregnant women with poor oral hygiene are more likely to develop gingivitis during pregnancy. | True | False | Not Sure |
| The second trimester is the best period for dental treatment. | True | False | Not Sure |
| Dental X-rays during pregnancy may affect fetal development. | True | False | Not Sure |
| It is safe during pregnancy to use fluoride toothpaste. | True | False | Not Sure |
| It is safe during pregnancy to use local anesthesia for dental treatment. | True | False | Not Sure |
| Acetaminophen is safe for pregnant women. | True | False | Not Sure |
| Ibuprofen is safe for pregnant women in the third trimester. | True | False | Not Sure |
| Penicillin antibiotics are safe for pregnant women. | True | False | Not Sure |
| Cephalosporin is safe for pregnant women. | True | False | Not Sure |
| Metronidazole is safe for pregnant women. | True | False | Not Sure |
| Maternal caries may promote caries in the child. | True | False | Not Sure |

1. **Practice of oral health care for pregnant women**

| Advise clinic attendees to address oral problems promptly before pregnancy. | Always | Sometimes | Never |
| --- | --- | --- | --- |
| Advice on referrals to dental specialists when pregnant women have oral problems. | Always | Sometimes | Never |
| Advise pregnant women to maintain oral hygiene during pregnancy. | Always | Sometimes | Never |
| Advise pregnant women to take oral examinations regularly. | Always | Sometimes | Never |
| Advise pregnant women to brush their teeth in the morning and evening. | Always | Sometimes | Never |
| Advise pregnant women to rinse their mouths after eating. | Always | Sometimes | Never |
| Advise pregnant women to use tooth flossing. | Always | Sometimes | Never |
| Advise pregnant women to brush their teeth with fluoride toothpaste. | Always | Sometimes | Never |
| Introduce the importance of maternal and newborn oral health care to pregnant women. | Always | Sometimes | Never |

1. **Barriers affecting antenatal care providers in providing oral health advice to pregnant women**

| Lack of time | Yes | No |
| --- | --- | --- |
| It’s the duty of dentists | Yes | No |
| Lack of knowledge about oral health care for pregnant women | Yes | No |
| Concerns about legal risks associated with negative birth outcomes | Yes | No |
| Lack of consensus with dentists on oral health care and treatment of pregnant women | Yes | No |
| Pregnant women are not interested or have no need. | Yes | No |

**产前保健提供者关于孕妇口腔保健的问卷调查**

**亲爱的参与者：**

您好！我们是本研究课题组成员，诚挚邀请您参与本项问卷调查。本研究旨在了解产前护理人员对孕妇口腔保健相关知识、态度及实践的现状，为后续制定科学、可行的健康教育和服务改进策略提供依据。本研究已通过伦理审查委员会审查，您的参与完全自愿。

1. 本问卷需根据您的实际情况和真实想法填写。填写过程中如有任何疑问，您可以随时向研究人员咨询。
2. 本研究为匿名问卷调查，不会对您的身体或心理造成伤害。问卷可能涉及您的性别、年龄、职业等基本信息，所有资料仅用于科学研究，并将严格保密。
3. 您有权随时了解与本研究相关的信息，也可以在问卷提交前自行退出。若您选择退出，相关数据将不会纳入研究分析。
4. 完成并提交问卷即表示您已阅读并理解上述说明，并同意参与本研究。

衷心感谢您在百忙之中支持本项科学研究！

□ 我已知晓并同意参与本研究，且同意所收集的数据用于科学研究。

1. **您的性别是：**

1）男性 2）女性

1. **您所处的年龄段是：**

1）≤30 2）31-40 3）41-50 4）>50

1. **您的最高学历是：**

1）专科 2）本科 3）硕士 4）博士

1. **您的职称是：**

1）初级 2）中级 3）高级

1. **您所在单位的级别是：**

1）一级 2）二级 3）三级

1. **您的岗位是：**

1）妇产科医生 2）护士 3）家庭医生

1. **您是否接受过孕妇口腔健康保健相关培训？**

1）从未 2）有时 3）总是

1. **您是否同意下述关于孕妇口腔保健的陈述？**
   1. 口腔保健应作为产前护理的一部分

1）完全同意 2）基本同意 3）中立 4）基本不同意 5）完全不同意

- 1. 孕妇口腔健康会影响婴儿口腔健康

1）完全同意 2）基本同意 3）中立 4）基本不同意 5）完全不同意

- 1. 产前护理人员有义务为孕妇提供孕妇和婴幼儿口腔保健建议

1）完全同意 2）基本同意 3）中立 4）基本不同意 5）完全不同意

- 1. 产前护理人员应该在门诊时为孕妇提供口腔保健建议

1）完全同意 2）基本同意 3）中立 4）基本不同意 5）完全不同意

- 1. 孕妇孕期出现口腔问题应推迟至分娩后处理

1）完全同意 2）基本同意 3）中立 4）基本不同意 5）完全不同意

**9、您认为下述说法正确吗？**

1. 孕妇的牙周疾病可能导致不良妊娠结局。

1）正确 2）错误 3）不确定

1. 怀孕期间孕妇的口腔卫生不佳更易患牙龈炎。

1）正确 2）错误 3）不确定

1. 孕中期是治疗孕妇口腔疾病的安全期。

1）正确 2）错误 3）不确定

1. 孕期接受口腔X线检查会影响胎儿发育。

1）正确 2）错误 3）不确定

1. 孕妇使用含氟牙膏是安全的。

1）正确 2）错误 3）不确定

1. 在孕妇常规口腔治疗中使用常规局麻药物是安全的。

1）正确 2）错误 3）不确定

1. 孕妇使用对乙酰氨基酚是安全的。

1）正确 2）错误 3）不确定

1. 孕妇在孕晚期使用布洛芬是安全的。

1）正确 2）错误 3）不确定

1. 孕妇使用青霉素类抗生素是安全的。

1）正确 2）错误 3）不确定

1. 孕妇使用头孢菌素类抗生素是安全的。

1）正确 2）错误 3）不确定

1. 孕妇使用甲硝唑是安全的。

1）正确 2）错误 3）不确定

1. 母亲患龋会提高孩子的患龋风险。

1）正确 2）错误 3）不确定

**10、您是否为孕妇提供过以下口腔保健服务：**

1. 建议就诊者怀孕前及时处理口腔问题

1）总是 2）偶尔 3）从不

1. 在孕妇遇到口腔问题时建议其到口腔专科就诊

1）总是 2）偶尔 3）从不

1. 建议孕妇在怀孕期间关注口腔健康

1）总是 2）偶尔 3）从不

1. 建议孕妇定期接受口腔检查

1）总是 2）偶尔 3）从不

1. 建议孕妇早晚刷牙

1）总是 2）偶尔 3）从不

1. 建议孕妇进食后漱口

1）总是 2）偶尔 3）从不

1. 建议孕妇使用牙线

1）总是 2）偶尔 3）从不

1. 建议孕妇使用含氟牙膏

1）总是 2）偶尔 3）从不

1. 向孕妇介绍产妇及新生儿口腔保健的重要性

1）总是 2）偶尔 3）从不

1. **您认为影响您为孕妇提供口腔保健的原因是什么？（可多选）**
2. 没时间
3. 是口腔医生的责任
4. 对孕妇口腔保健相关知识缺乏了解
5. 担心与不良妊娠结局相关的法律风险
6. 与口腔医生在孕妇口腔保健和治疗方面缺乏共识
7. 孕妇不感兴趣或没有需求
