# Supplementary material for: Knowledge, attitude, and practice of antenatal care providers about oral health care for pregnant women: a cross-sectional survey study in Shanghai
Source: Front Public Health. 2026 Jun 22;14:1807833. doi: 10.3389/fpubh.2026.1807833 (PMC13333608; doi:10.3389/fpubh.2026.1807833)
Supplement: Supplementary file 2 [file Table_1.docx]

**Supplementary Table S1**. Comparison of KAP scores among different demographic characteristics classification in OB-GYNs.

| Index | OB-GYN (n=115), M (IQR) | | | | | |
| --- | --- | --- | --- | --- | --- | --- |
|  | Knowledge scores | P | Attitude scores | P | Practice scores | P |
| Gender |  |  |  |  |  |  |
| Male | 8.5 (4) | 0.513 | 23 (3) | 0.011 | 25 (5) | 0.238 |
| Female | 9 (3) |  | 21 (3) |  | 23 (6) |  |
| Age (years) |  |  |  |  |  |  |
| ≤30 | 9 (3) | 0.223 | 21 (4) | 0.040 | 20 (7) | 0.021 |
| 31-40 | 9 (2) |  | 21 (3) |  | 25 (6) |  |
| 41-50 | 9 (3) |  | 22 (2) |  | 23 (3) |  |
| >50 | 7 (4) |  | 23 (4) |  | 24.5 (6) |  |
| Education |  |  |  |  |  |  |
| Associate & Bachelor | 8 (3) | <0.001 | 21 (3) | 0.587 | 24 (6) | 0.220 |
| Master | 9 (2) |  | 22 (4) |  | 24 (6) |  |
| PhD | 10 (1) |  | 22 (2) |  | 23 (7) |  |
| Title |  |  |  |  |  |  |
| Residency | 9 (3) | 0.409 | 22 (3) | 0.906 | 24 (5) | 0.927 |
| Attending | 9 (2) |  | 21 (3) |  | 24 (7) |  |
| Professor | 9 (4) |  | 21 (3) |  | 23 (7) |  |
| Hospital level |  |  |  |  |  |  |
| Secondary | 8 (4) | 0.286 | 21 (3) | 0.997 | 25 (3) | 0.114 |
| Tertiary | 9 (3) |  | 22 (3) |  | 23 (6) |  |
| Training experience |  |  |  |  |  |  |
| Never | 9 (3) | 0.147 | 22 (4) | 0.805 | 21.5 (6) | <0.001 |
| Sometimes | 8 (4) |  | 21 (3) |  | 25 (4) |  |
| Always | 9 (3) |  | 21 (2) |  | 27 (2) |  |

All data are shown as median (interquartile range, IQR).

KAP, knowledge, attitude, and practice; IQR, interquartile range.

Training experience was based on self-reported previous professional training related to oral health care for pregnant women and was categorized as never, sometimes, or always.

Knowledge score was calculated from 12 items, with 1 point assigned for each correct answer and 0 points assigned for an incorrect or “not sure” response.

Attitude score was derived from five items assessing attitudes toward oral health care during pregnancy, each scored on a five-point Likert scale from 1 to 5. The total score ranges 5-25, and higher total scores indicate more positive attitudes.

Practice score was derived from 9 items assessing self-reported oral health care practices for pregnant women. The total score ranges 9-27, and higher scores indicate better practice.

**Supplementary Table S2.** Comparison of KAP scores among different demographic characteristics classification in family physicians.

| Index | Family physicians (n=110), M (IQR) | | | | | |
| --- | --- | --- | --- | --- | --- | --- |
|  | Knowledge scores | P | Attitude scores | P | Practice scores | P |
| Gender |  |  |  |  |  |  |
| Male | 7 (5) | 0.488 | 21 (4) | 0.879 | 24 (5) | 0.778 |
| Female | 5 (3) |  | 21 (4) |  | 25 (5) |  |
| Age (years) |  |  |  |  |  |  |
| ≤30 | 6 (4) | 0.964 | 20 (4) | 0.046 | 21.5 (9) | 0.453 |
| 31-40 | 5 (4) |  | 21 (4) |  | 23.5 (6) |  |
| 41-50 | 5.5 (4) |  | 21 (3) |  | 25 (4) |  |
| ＞50 | 5 (6) |  | 23 (3) |  | 25 (3) |  |
| Education |  |  |  |  |  |  |
| Associate & Bachelor | 5 (5) | 0.031 | 21 (4) | 0.621 | 25 (5) | 0.436 |
| Master | 7 (4) |  | 21 (4) |  | 23 (6) |  |
| Title |  |  |  |  |  |  |
| Residency | 5 (3) | 0.834 | 21 (6) | 0.280 | 23 (10) | 0.735 |
| Attending | 5 (4) |  | 21 (3) |  | 24 (5) |  |
| Professor | 7 (5) |  | 21.5 (3) |  | 25 (4) |  |
| Training experience |  |  |  |  |  |  |
| Never | 5 (3) | 0.003 | 21 (4) | 0.751 | 23 (5) | <0.001 |
| Sometimes | 6 (5) |  | 21 (4) |  | 25 (4) |  |
| Always | 8 (2) |  | 21 (0) |  | 27 (1) |  |

All data are shown as median (interquartile range, IQR).

KAP, knowledge, attitude, and practice; IQR, interquartile range.

Training experience was based on self-reported previous professional training related to oral health care for pregnant women and was categorized as never, sometimes, or always.

Knowledge score was calculated from 12 items, with 1 point assigned for each correct answer and 0 points assigned for an incorrect or “not sure” response.

Attitude score was derived from five items assessing attitudes toward oral health care during pregnancy, each scored on a five-point Likert scale from 1 to 5. The total score ranges 5-25, and higher total scores indicate more positive attitudes.

Practice score was derived from 9 items assessing self-reported oral health care practices for pregnant women. The total score ranges 9-27, and higher scores indicate better practice.

**Supplementary Table S3.** Comparison of KAP scores among different demographic characteristics classification in nurses

| Index | Nurses (n=234), M (IQR) | | | | | |
| --- | --- | --- | --- | --- | --- | --- |
|  | Knowledge scores | P | Attitude scores | P | Practice scores | P |
| Gender |  |  |  |  |  |  |
| Male | 6 (5) | 0.584 | 21 (6) | 0.837 | 24 (6) | 0.921 |
| Female | 6 (4) |  | 21 (4) |  | 23 (6) |  |
| Age (years) |  |  |  |  |  |  |
| ≤30 | 6 (3) | <0.001 | 21 (4) | 0.008 | 23 (7) | 0.745 |
| 31-40 | 7 (4) |  | 21 (4) |  | 24 (6) |  |
| 41-50 | 5 (4) |  | 23 (4) |  | 23.5 (5) |  |
| ＞50 | 4 (3) |  | 21 (5) |  | 22 (6) |  |
| Education |  |  |  |  |  |  |
| Associate | 6 (5) | 0.249 | 21 (4) | 0.088 | 22 (6) | 0.155 |
| Bachelor | 6 (4) |  | 21 (4) |  | 24 (6) |  |
| Master | 6 (2) |  | 19 (3) |  | 26 (8) |  |
| Title |  |  |  |  |  |  |
| Residency | 6 (4) | 0.725 | 21 (4) | 0.389 | 22 (5) | 0.922 |
| Attending | 6 (4) |  | 21 (4) |  | 23 (8) |  |
| Professor | 6 (4) |  | 21 (6) |  | 24 (5) |  |
| Hospital level |  |  |  |  |  |  |
| Secondary | 5 (5) | 0.020 | 21 (2) | 0.417 | 25 (2) | 0.003 |
| Tertiary | 6 (4) |  | 21 (4) |  | 22 (6) |  |
| Training experience |  |  |  |  |  |  |
| Never | 6 (3) | 0.037 | 21 (4) | 0.868 | 22 (7) | <0.001 |
| Sometimes | 6 (5) |  | 21 (4) |  | 24 (5) |  |
| Always | 8 (3) |  | 21 (0) |  | 26 (2) |  |

All data are shown as median (interquartile range, IQR).

KAP, knowledge, attitude, and practice; IQR, interquartile range.

Training experience was based on self-reported previous professional training related to oral health care for pregnant women and was categorized as never, sometimes, or always.

Knowledge score was calculated from 12 items, with 1 point assigned for each correct answer and 0 points assigned for an incorrect or “not sure” response.

Attitude score was derived from five items assessing attitudes toward oral health care during pregnancy, each scored on a five-point Likert scale from 1 to 5. The total score ranges 5-25, and higher total scores indicate more positive attitudes.

Practice score was derived from 9 items assessing self-reported oral health care practices for pregnant women. The total score ranges 9-27, and higher scores indicate better practice.

**Supplementary Table S4.** Antenatal care providers’ knowledge about oral health during pregnancy

| Index | Correct  n (%) | | | Chi-squared/H | P |
| --- | --- | --- | --- | --- | --- |
|  | OB-GYN (n=115) | Family physicians (n=110) | Nurses (n=234) |  |  |
| Periodontal disease in pregnant women may lead to adverse pregnancy outcomes. (YES) | 92 (80.0) | 75 (68.2) | 131 (56.0) | 20.205 | <0.001 |
| Pregnant women with poor oral hygiene are more likely to develop gingivitis during pregnancy. (YES) | 109 (94.8) | 105 (95.5) | 217 (92.7) | 1.175 | 0.563 |
| The second trimester is the best period for dental treatment. (YES) | 89 (77.4) | 67 (60.9) | 118 (50.4) | 23.389 | <0.001 |
| Dental X-rays during pregnancy may affect fetal development. (NO) | 70 (60.9) | 22 (20.0) | 42 (17.9) | 74.633 | <0.001 |
| It is safe during pregnancy to use fluoride toothpaste. (YES) | 52 (45.2) | 24 (21.8) | 66 (28.2) | 16.075 | <0.001 |
| It is safe during pregnancy to use local anesthesia for dental treatment. (YES) | 108 (93.9) | 68 (61.8) | 146 (62.4) | 41.384 | <0.001 |
| Acetaminophen is safe for pregnant women. (YES) | 78 (67.8) | 22 (20.0) | 77 (32.9) | 60.726 | <0.001 |
| Ibuprofen is safe for pregnant women in the third trimester. (NO) | 17 (14.8) | 34 (30.9) | 46 (19.7) | 9.396 | 0.009 |
| Penicillin antibiotics are safe for pregnant women. (YES) | 109 (94.8) | 69 (62.7) | 162 (69.2) | 35.913 | <0.001 |
| Cephalosporin is safe for pregnant women. (YES) | 108 (93.9) | 49 (44.5) | 144 (61.5) | 64.150 | <0.001 |
| Metronidazole is safe for pregnant women. (YES) | 68 (59.1) | 16 (14.5) | 91 (38.9) | 47.493 | <0.001 |
| Maternal caries may promote caries in the child. (YES) | 77 (67.0) | 70 (63.6) | 136 (58.1) | 2.787 | 0.248 |
| Score (M(IQR)) | 9 (3) | 5 (4) | 6 (4) | 84.688 | <0.001 |

All data are shown as n (%).

OB-GYN, obstetrician-gynecologist.

For each statement, the correct answer is shown in parentheses.

**Supplementary Table S5.** Antenatal care providers’ attitude about oral health care for pregnant women

| Index | OB-GYN (n=115) | | | Family physicians (n=110) | | | Nurses (N=234) | | | Chi-squared | P |
| --- | --- | --- | --- | --- | --- | --- | --- | --- | --- | --- | --- |
|  | Agree | Neutral | Disagree | Agree | Neutral | Disagree | Agree | Neutral | Disagree |  |  |
| Oral health care should be part of prenatal care. | 111  (96.5) | 4 (3.5) | 0 (0) | 107  (97.3) | 3 (2.7) | 0 (0) | 220  (94.0) | 13  (5.6) | 1 (0.4) | 4.835 | 0.571* |
| Maternal oral health affects infant oral health. | 81  (70.4) | 28 (24.3) | 6 (5.2) | 99  (90.0) | 9 (8.2) | 2 (1.8) | 192  (82.1) | 34  (14.5) | 8 (3.4) | 14.974 | 0.040* |
| Antenatal care providers should advise pregnant women on maternal and infant oral health care information about maternal and infant. | 105  (91.3) | 8 (7.0) | 2 (1.7) | 97  (88.2) | 13 (11.8) | 0 (0) | 208  (88.9) | 22  (9.4) | 4 (1.7) | 6.006 | 0.402* |
| Antenatal care providers should provide oral health advice to pregnant women at clinic visits. | 106  (92.2) | 7 (6.1) | 2 (1.7) | 96  (87.3) | 14  (12.7) | 0 (0) | 210  (89.7) | 23  (9.8) | 1 (0.4) | 5.203 | 0.488* |
| Oral problems during pregnancy should be postponed until after delivery. | 34  (29.6) | 28 (24.3) | 53 (46.1) | 52  (47.3) | 27 (24.5) | 31 (28.2) | 101  (43.2) | 59  (25.2) | 74 (31.6) | 14.584 | 0.068 |

All data are shown as n (%).

*: Fisher's exact test

OB-GYN: obstetricians and gynecologists.

**Supplementary Table S6.** Antenatal care providers’ practice of oral health care for pregnant women

| Index | OB-GYN (n=115) | | | Family physicians (n=110) | | | Nurses (n=234) | | | Chi-squared/H | P |
| --- | --- | --- | --- | --- | --- | --- | --- | --- | --- | --- | --- |
|  | Always | Sometimes | Never | Always | Sometimes | Never | Always | Sometimes | Never |  |  |
| Advise clinic attendees to address oral problems promptly before pregnancy. | 71 (61.7) | 32 (27.8) | 12 (10.4) | 67 (60.9) | 27 (24.5) | 16 (14.5) | 119 (50.9) | 88 (37.6) | 27 (11.5) | 7.959 | 0.093 |
| Advice on referrals to dental specialists when pregnant women have oral problems. | 96 (83.5) | 16 (13.9) | 3 (2.6) | 101(91.8) | 6 (5.5) | 3 (2.7) | 158 (67.5) | 68 (29.1) | 8 (3.4) | 32.683 | <0.001* |
| Advise pregnant women to maintain oral hygiene during pregnancy. | 76 (66.1) | 30 (26.1) | 9 (7.8) | 84 (76.4) | 19 (17.3) | 7(6.4) | 149 (63.7) | 76 (32.5) | 9 (3.8) | 10.717 | 0.030 |
| Advise pregnant women to take oral examinations regularly. | 62 (53.9) | 39 (33.9) | 14 (12.2) | 83 (75.5) | 22 (20.0) | 5 (4.5) | 132 (56.4) | 80 (34.2) | 22 (9.4) | 14.743 | 0.005 |
| Advise pregnant women to brush their teeth in the morning and evening. | 96 (83.5) | 14 (12.2) | 5 (4.3) | 100 (90.9) | 7 (6.4) | 3 (2.7) | 197 (84.2) | 30 (12.8) | 7 (3.0) | 4.195 | 0.376* |
| Advise pregnant women to rinse their mouths after eating. | 81 (70.4) | 27 (23.5) | 7 (6.1) | 91 (82.7) | 14 (12.7) | 5 (4.5) | 181 (77.4) | 42 (17.9) | 11 (4.7) | 5.074 | 0.280 |
| Advise pregnant women to use tooth flossing. | 58 (50.4) | 40 (34.8) | 17 (14.8) | 71 (64.5) | 25 (22.7) | 14 (12.7) | 123 (52.6) | 71 (30.3) | 40 (17.1) | 6.323 | 0.176 |
| Advise pregnant women to brush their teeth with fluoride toothpaste. | 33 (28.7) | 38 (33.0) | 44 (38.3) | 27 (24.5) | 27 (24.5) | 56 (50.9) | 63 (26.9) | 75 (32.1) | 96 (41.0) | 4.492 | 0.344 |
| Introduce the importance of maternal and newborn oral health care to pregnant women. | 52 (45.2) | 43 (37.4) | 20 (17.4) | 67 (60.9) | 28 (25.5) | 15 (13.6) | 122 (52.1) | 82 (35.0) | 30 (12.8) | 6.636 | 0.156 |
| Score (M (IQR)) | 24 (6) | | | 24 (5) | | | 23 (6) | | | 4.638 | 0.098 |

All data are shown as n (%) or median (interquartile range).

*: Fisher's exact test

OB-GYN: obstetricians and gynecologists.
